# Suppression of CEBPδ recovers exhaustion in anti-metastatic immune cells

**DOI:** 10.1038/s41598-023-30476-4

**Published:** 2023-03-08

**Authors:** Chenxue Yin, Masayoshi Kato, Takeshi Tomita, Yibing Han, Sachie Hiratsuka

**Affiliations:** 1 Institute for Biomedical Sciences, Interdisciplinary Cluster for Cutting Edge Research, Shinshu University, School of Medicine, 3-1-1 Asahi, Matsumoto, Nagano 390-8621 Japan; 2 Department of Biochemistry and Molecular Biology, Shinshu University, School of Medicine, 3-1-1 Asahi, Matsumoto, Nagano 390-8621 Japan

**Keywords:** Metastasis, Tumour immunology

## Abstract

The pre-metastatic microenvironment consists of pro-metastatic and anti-metastatic immune cells in the early stages of cancer, when the primary tumor begins to proliferate. Redundantly, pro-inflammatory immune cells predominated during tumor growth. Although it is well known that pre-metastatic innate immune cells and immune cells fighting primary tumor cells become exhausted, the mechanism by which this occurs is unknown. We discovered that anti-metastatic NK cells were mobilized from the liver to the lung during primary tumor progression and that the transcription factor CEBPδ, which was upregulated in a tumor-stimulated liver environment, inhibited NK cell attachment to the fibrinogen-rich bed in pulmonary vessels and sensitization to the environmental mRNA activator. CEBPδ-siRNA treated anti-metastatic NK cells regenerated the binding proteins that support sitting in fibrinogen-rich soil, such as vitronectin and thrombospondin, increasing fibrinogen attachment. Furthermore, CEBPδ knockdown restored an RNA-binding protein, ZC3H12D, which captured extracellular mRNA to increase tumoricidal activity. Refreshed NK cells using CEBPδ-siRNA with anti-metastatic abilities would work at metastatic risk areas in the pre-metastatic phase, resulting in a reduction in lung metastasis. Furthermore, tissue-specific siRNA-based therapy in lymphocyte exhaustion may be beneficial in the treatment of early metastases.

## Introduction

Primary tumor growth includes multiple steps, including hyperplasia, adenoma, carcinoma in situ, and advanced carcinoma. Aside from that, metastasis begins at an early stage^1^. During tumor metastasis, two different events in tumor cells and host microenvironment, tumor cell dissemination and pre-metastatic soil preparation occur independently. The term “pre-metastatic niche” refers to an immune cell mobilization site by the primary tumor in a distant organ before the arrival of primary tumor cells, and it has been studied in mouse models^2–13^. These studies revealed that migrating immune cells, like tissue-resident cells, contribute to the pre-metastatic soil, and their roles are delicately controlled by many factors. Indeed, migrating immune cells in the lungs can either attack or support metastatic tumor cells invading the lungs^11,14^. It is well known that bone marrow (BM) contains a significant amount of migrating immune cells ^15^. Thus, in the case of lung metastasis, the primary tumor, lung, and BM form a metastatic triangle. Furthermore, a recent study discovered that the liver, which was previously thought to be a bystander in the metastatic triangle, played an important role in pre-metastatic soil formation in the lung^16^. According to a mouse model study, anti-metastatic NK cells were educated in the liver affected by the primary tumor and then moved to the lungs. Anti-metastatic NK cells were capable of relocating the pre-metastatic soil through the expression of vitronectin (Vtn) or thrombospondin (Tsp) on the cell surface. Furthermore, the extracellular RNA-ZC3H12D signaling axis sensitized these cells to increase tumoricidal activity^17^. There is a growing expectation that clinical applications of NK cells will become more common because NK cells have a high potential ability for attacking tumor cells^18^. However, a recent study of tumor-associated NK cells in patients found that NK cells in the tumor microenvironment had low anti-tumor activity^19^. This data suggested that some external support may be required to maintain NK cells' anti-tumor or anti-metastatic activity. In this study, we tested a molecular intervention to liver-educated anti-metastatic NK cells, because these NK cells also showed exhaustion, or a decrease in anti-tumor activity, in our mouse model study. We first screened NK cell transcription factors to determine that CEBPδ is a key transcription factor and demonstrated that knockdown of CEBPδ made NK cells into more anti-tumor character, implying that blocking CEBPδ prevents NK cell exhaustion.

## Results

### Expression of fibrinogen binding molecules in a tumor-bearing state

**Figure 1:**
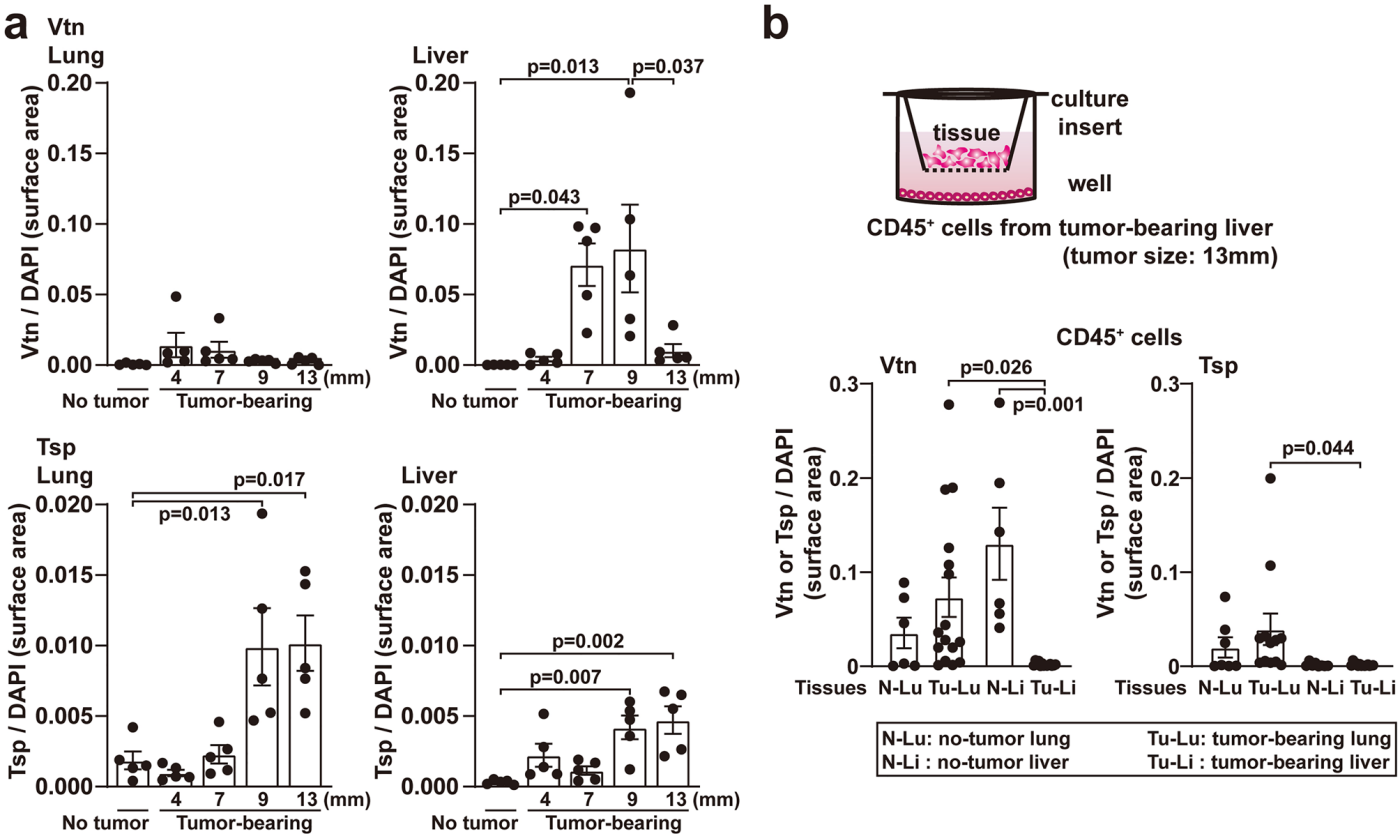
Vitronectin and thrombospondin 1 expression in tumor-bearing lungs and liver. (**a**) Unique expression of vitronectin (Vtn) and increase of thrombospondin 1 (Tsp) in tumor-bearing liver and lungs, respectively. Protein levels were examined immunohistochemically and normalized with DAPI. Tumor sizes which were the average of vertical and horizontal length in the maximum transverse section, are demonstrated in the graph. N = 4–5. (**b**) In vitro assay system for CD45^+^ cells from tumor-bearing livers cultured with conditioned media (CM), with organ tissues in separate culture inserts. The protein expression levels were evaluated immunohistochemically and normalized with DAPI. CM was derived from tumor-free lung (N-Lu), tumor-free liver (N-Li), tumor-bearing lung (Tu-Lu), or tumor-bearing liver (Tu-Li) cultures. Primary tumor diameter: 13 mm. N = 3–7 (2 tissue lobes in each mouse). Error bars indicate mean ± SEM, and *P* values are indicated in graphs (**a**, **b**).

We immunohistochemically assessed Vtn-Tsp expression patterns in the liver and lungs, where tumor-associated leukocytes were mobilized during primary tumor progression. Vtn expression in the liver was upregulated and then downregulated during primary tumor development, whereas Tsp expression was upregulated in the lungs (Fig. 1a and Supplementary Fig. S1a). We discovered that using a tissue culture system in which tumor-bearing mice’s liver CD45^+^ leukocytes were cultured in the lower wells and various diced tissues were seeded in the upper culture insert, the tissue heavily influenced Vtn and Tsp expressions (Fig. 1b). CD45^+^ cells cocultured with normal liver tissue appeared to induce Vtn expression more than tumor-bearing liver (Fig. 1b). Liver tissue bearing earlier-stage tumors (~ 9 mm size) stimulated Vtn expression (Fig. 1a), whereas it was suppressed in liver tissue bearing progressed-stage tumors (~ 13 mm size) (Fig. 1a, b). Alternatively, tumor-bearing lung tissue tended to induce Tsp expression (Fig. 1b). To further investigate the protein expression pattern of Vtn and Tsp in single liver CD45^+^ cells stimulated by the liver or lung tissue, we collected CD45^+^ cells from tumor-bearing mouse livers and cultured them with tumor-bearing liver or lung tissue (Supplementary Fig. S2a). When lung-conditioned media (CM) from tumor-bearing mice stimulated liver CD45^+^ cells, Vtn expression was downregulated while Tsp expression was upregulated. However, Vtn expression was high following coculture with liver CM (Supplementary Fig. S2b).

### Vtn and CEBPδ showed inverse expression patterns in tumor-bearing liver CD45^+^ cells in the pre-metastatic phase

**Figure 2:**
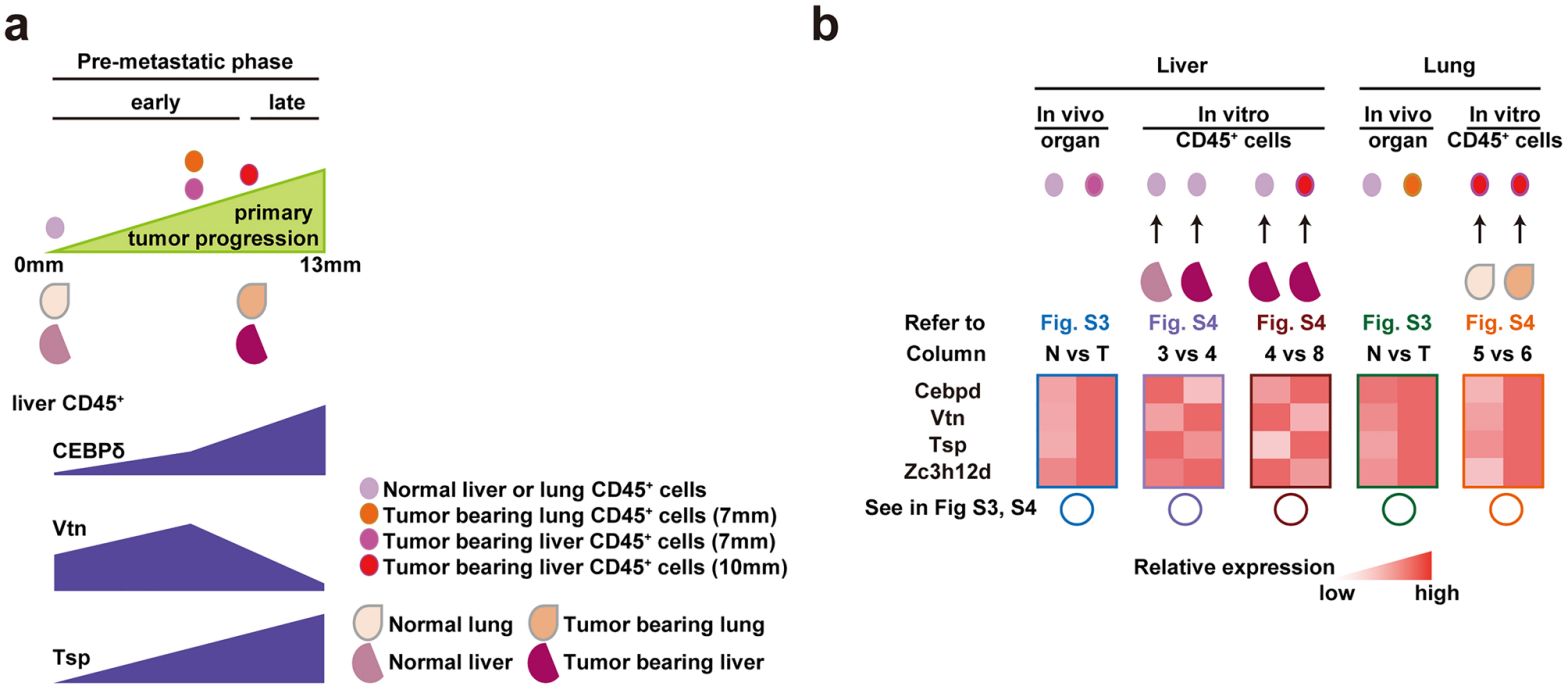
Expression pattern relationships among Vtn, Tsp, Zc3h12d, and CEBPδ in liver CD45^+^ cells. (**a**) Timeline of inverse correlation between Vtn and Tsp, regulated by CEBPδ in tumor-bearing organs. In the early pre-metastatic phase, the primary tumor moderately upregulates CEBPδ and Vtn in liver CD45^+^ cells. In the late pre-metastatic phase, the growing primary tumor suppressed Vtn by highly expressed CEBPδ. Detached liver CD45^+^ cells journey to the lungs, inducing Tsp upregulation. (**b**) Heatmaps of the positive correlation between CEBPδ and Vtn in early non-metastatic liver tissue from a tumor-bearing mouse, based on the in vivo data shown in Supplementary Fig. S3 (blue circles), in which mRNA levels of Vtn, Tsp, ZC3H12D, and multiple liver-related transcription factors in organs obtained from tumor-free or E0771-bearing (tumor size; 7 mm) Color coded illustrations depict CD45^+^ cells, lung, or liver tissues depicted in (**a**). Reverse expression of CEBPδ and Vtn in liver CD45^+^ cells activated by tumor-free or tumor-bearing liver tissue, based on the in vitro data shown in Supplementary Fig. S4 (3 vs 4, light purple circles) in which mRNA levels in CD45^+^ cells stimulated by organ tissues derived from tumor-free or E0771-bearing (tumor size; 10 mm). If tumor-bearing (10 mm) liver CD45^+^ cells were stimulated by tumor-bearing livers, the expression pattern of CEBPδ and Vtn was inverse (4 vs 8, red–purple in Supplementary Fig. S4).

We discovered liver-specific transcription factors that are activated in tumor-bearing states to regulate Vtn and Tsp expression in liver CD45^+^ cells. Several transcription factors regulate the liver’s marker gene expression, cellular function, and morphology^20–22^. Therefore, we chose 11 candidates that met these criteria from the gene expression arrays, as well as several proteins that could act as transcriptional regulators of Vtn and Tsp. Our promoter sequence analysis revealed that liver-specific transcription factors are involved in the regulation of Vtn and/or Tsp expression, such as Hnf1α CEBPα and CEBPδ for Vtn, Ap2a for Tsp, and Hnf3α (Foxa1), Hnf3β (Foxa2), Sp1, Gata6, and NF-κB (Rela) for both. Furthermore, based on our array data, Hnf4α and Foxa3 showed strong expression in liver CD45^+^ cells (Supplementary Table S1). The pre-metastatic phase can be divided into two phases based on Vtn expression levels, as shown in Fig. 2a. In the early pre-metastatic phase, the primary tumor is relatively small (~ 10 mm), but in the late pre-metastatic phase, it grows larger (~ 13 mm). As shown later, the effects of the primary tumor in distant organs vary depending on the size of the primary tumor. Furthermore, leukocyte migration ability was increased or decreased depending on primary tumor size. The expression profiles of lung CD45^+^ cells, liver CD45^+^ cells, lung tissues, and liver tissues (illustrated in Fig. 2a) are summarized as heatmaps in Fig. 2b, and these data clearly show that CEBPδ expression may contribute to Vtn, Tsp, and ZC3H12D expression. We focused on transcription factor genes that differed by more than 1.5-fold between no tumor-bearing and tumor-bearing liver (detailed data in Supplementary Fig. S3 and S4). The increase in Vtn and Tsp observed in the early pre-metastatic liver was consistent with the upregulation of CEBPδ, and Tsp expression enhanced in the lungs (blue and green in Fig. 2b; more detailed, blue, and green circles in Supplementary Fig. S3). We created various combinations using CD45^+^ cells isolated from the liver and lungs of mice with/without tumors. These experimental combinations were used to evaluate the specific relationships between transcription factors and Vtn-Tsp expression levels in liver CD45^+^ cells (see the light purple and red purple circles in Fig. 2b and Supplementary Fig. S4). Vtn expression in CD45^+^ cells was liver-specific (Supplementary Fig. S4, Vtn). In normal liver CD45^+^ cells, Vtn expression was more induced by coculture with tumor-bearing liver tissue than by coculture with tumor-free liver tissue (No 3 and No 4 in Vtn in Fig. 2b). Tumor-bearing liver tissue isolated in the late pre-metastatic phase suppressed Vtn expression more strongly in tumor-bearing CD45^+^ cells than in tumor-free CD45^+^ cells (No 4 and No 8 in Vtn in Fig. 2b). In extreme cases, Vtn expression may have been suppressed in tumor-bearing liver CD45^+^ cells (Fig. 1a, b). Interestingly, CEBPδ and Vtn expression were inversely related to CD45^+^ cells in a tumor growth-dependent manner (No 3, 4, and 8 in CEBPδ in Fig. 2b). Thus, CEBPδ in the pre-metastatic phase may regulate Vtn expression in liver CD45^+^ cells (Fig. 2b). In terms of Tsp expression, liver CD45^+^ cells derived from tumor-bearing mice showed increased expression when stimulated with tumor-baring lungs (orange circles No 5 and 6 in Fig. 2b and Supplementary Fig. S4). Recently, we discovered that anti-metastatic immune cells in the liver CD45^+^ cells expressed an RNA-binding protein, ZC3H12D, on the cell surface, and its binding ligand is a nonvesicular-type mRNA^17^. ZC3H12D expression was similar to Vtn in liver CD45^+^ cells (Fig. 2b).

### CEBPδ controls Vtn-Tsp expression and migration of liver CD45^+^ cells from tumor-bearing mice to the lungs

**Figure 3:**
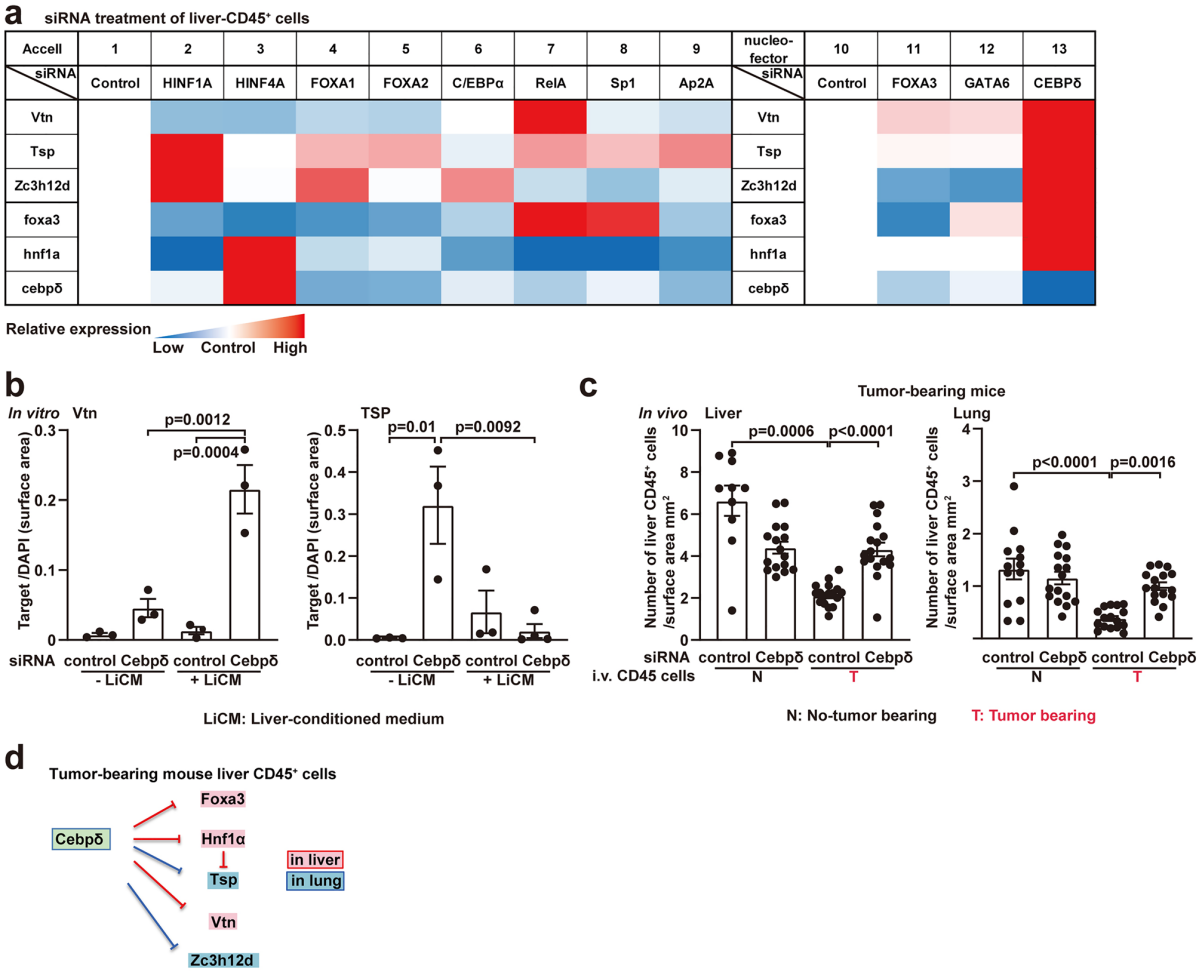
Primary tumor-stimulating CEBPδ regulates Vtn, Tsp, and ZC3H12D in the tumor-bearing liver and lungs. (**a**) Heatmap of mRNA levels in liver CD45^+^ cells treated with siRNA based on Supplementary Fig. S6. One of the two distinct siRNA delivery systems was utilized for each siRNA (transfection “Accell” and electroporation “Nucleofector”) and evaluated with control (nontarget) siRNA. (**b**) Protein levels of Vtn and Tsp in siRNA-treated tumor-bearing liver CD45^+^ cells cultured with/without a liver-conditioned medium (LiCM) derived from tumor-bearing mice. DAPI normalized immunohistochemical signals. N = 3. (**c**) Number of accumulated siRNA-treated liver CD45^+^ cells in the liver (left) and lung (right) after injection in E0771-bearing recipient mice. Red words indicate substantial results. N = 8 (1–2 tissue lobes in each mouse). Error bars indicate the mean ± SEM, and *P* values (significant) are indicated in graphs (**a**–**c**). (**d**) Summary of CEBPδ inhibitory regulation in tumor-bearing mouse liver CD45^+^ cells using an in vitro siRNA assay, per the data shown in Supplementary Fig. S6.

CEBPδ has a multifaceted role in tumor progression, acting as both a suppressor and a promoter^23–25^. To better understand the roles of CEBPδ in controlling CD45^+^ cells in the pre-metastatic phase, we performed gene knockdown experiments for the above-noted 11 transcription factors, including CEBPδ. We began by looking for the transcription factor that regulates the expression of Vtn and Tsp. Using siRNAs validated for each molecule in liver CD45^+^ cells (Supplementary Fig. S5), we tested their effects on the expression of Vtn-Tsp in tumor-bearing liver CD45^+^ cells (Fig. 3a and Supplementary Fig. S6). We discovered that CEBPδ knocked down tended to increase the expression of Vtn, Tsp, ZC3H12D, Hnf1α, and Foxa3 (Fig. 3a, d). Furthermore, Hnf1α-siRNA treatment increased Tsp expression, indicating that Hnf1α was involved in the regulation of Tsp (Fig. 3a, d). To investigate the effect of the liver environment on Vtn and Tsp expression, we cultured CEBPδ-siRNA-treated tumor-bearing liver CD45^+^ cells in a liver-conditioned medium (LiCM). As expected, CEBPδ-siRNA treatment increased Vtn and Tsp levels in CD45^+^ liver cells without LiCM (Fig. 3b). However, Tsp was downregulated in the presence of tumor-bearing LiCM following CEBPδ-siRNA administration, despite Vtn being upregulated under the same conditions (Fig. 3b). Tissue-specific factors may collaborate with Tsp, although CEBPδ regulates Vtn and Tsp expression. The homing ability of CD45^+^ liver cells in liver and lung tissue was then investigated with CEBPδ knocked down. Following CEBPδ-siRNA treatment, fluorescently labeled liver CD45^+^ cells from tumor-free and tumor-bearing mice were injected into the tail veins of individual mice, and CD45^+^ cell accumulation in the liver and lungs was quantified. Figure 3c depicts a clear decrease in tumor-bearing liver CD45^+^ cells in tumor-bearing mice (column 1 *vs*. 3). This finding implies that the primary tumor significantly impaired liver CD45^+^ cells’ homing capacity to both the lung and liver. Finally, the recovery of CD45^+^ accumulation was observed in tumor-bearing lungs and livers after CEBPδ-siRNA treatment (column 4 in Fig. 3c).

### CEBPδ-siRNA-treated CD45^+^ liver cells suppress lung metastasis

**Figure 4:**
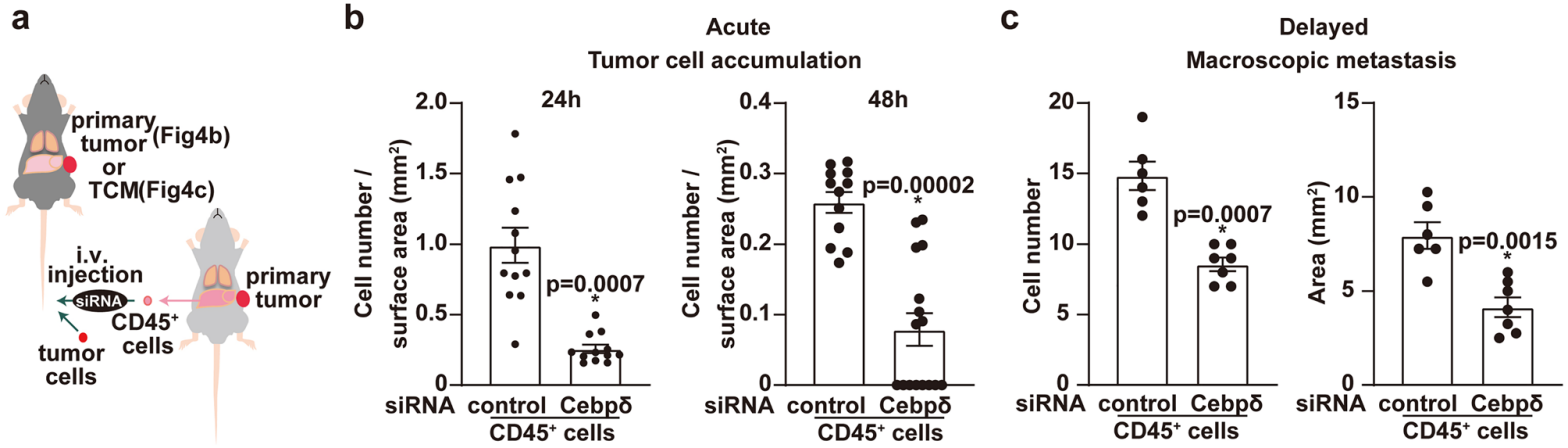
Reduction of lung metastasis by CEBPδ-siRNA-treated liver CD45^+^ cells in tumor-bearing mice. (**a**) Experimental scheme of the intravenous (i.v.) injection of tumor cells into tumor-bearing mice (**b**) and TCM-stimulated mice (**c**). (**b**) E0771 cell homing after injection of siRNA-treated tumor-bearing liver CD45^+^ cells. Numbers of tumor cells in lungs 24 h (left) and 48 h (right) after tumor cell injection into tumor-bearing WT mice. N = 6, (two lobes in each group). (**c**) Number of macroscopic metastatic nodules three weeks after siRNA-treated tumor-bearing liver CD45^+^ cell treatment followed by injection of tumor cells into tumor-conditioned medium (TCM)-stimulated mice. N = 6–7. Error bars represent the mean ± SEM, and *P* values (significant) are shown in graphs (**a**, **b**).

To protect against lung metastasis, we attempted to generate liver CD45^+^ cells with efficient homing ability, via Vtn, and Tsp, using CEBPδ-siRNA. After treating tumor-bearing liver CD45^+^ cells with CEBPδ-siRNA, we pre-injected these cells intravenously into tumor-bearing mice, followed by injection of fluorescent-labeled tumor cells (Fig. 4a, experimental model). The number of homing tumor cells in tumor-bearing lungs decreased significantly after the pre-infusion of CEBPδ-siRNA-treated liver CD45^+^ cells derived from tumor-bearing mice compared to control siRNA-treated cells (Fig. 4b). Conversely, the accumulation of CEBPδ-siRNA-treated liver CD45^+^ cells in the lungs was greater than that of control siRNA-treated cells (Supplementary Fig. S7). To assess whether acute tumor colonization contributes to macroscopic metastasis, we observed metastasis in TCM-stimulated mice 3–4 weeks after the application of siRNA-treated liver CD45^+^ cells followed by tumor cell injection. A macroscopic metastasis analysis yielded similar results to acute metastasis (Fig. 4b, c).

**Figure 5:**
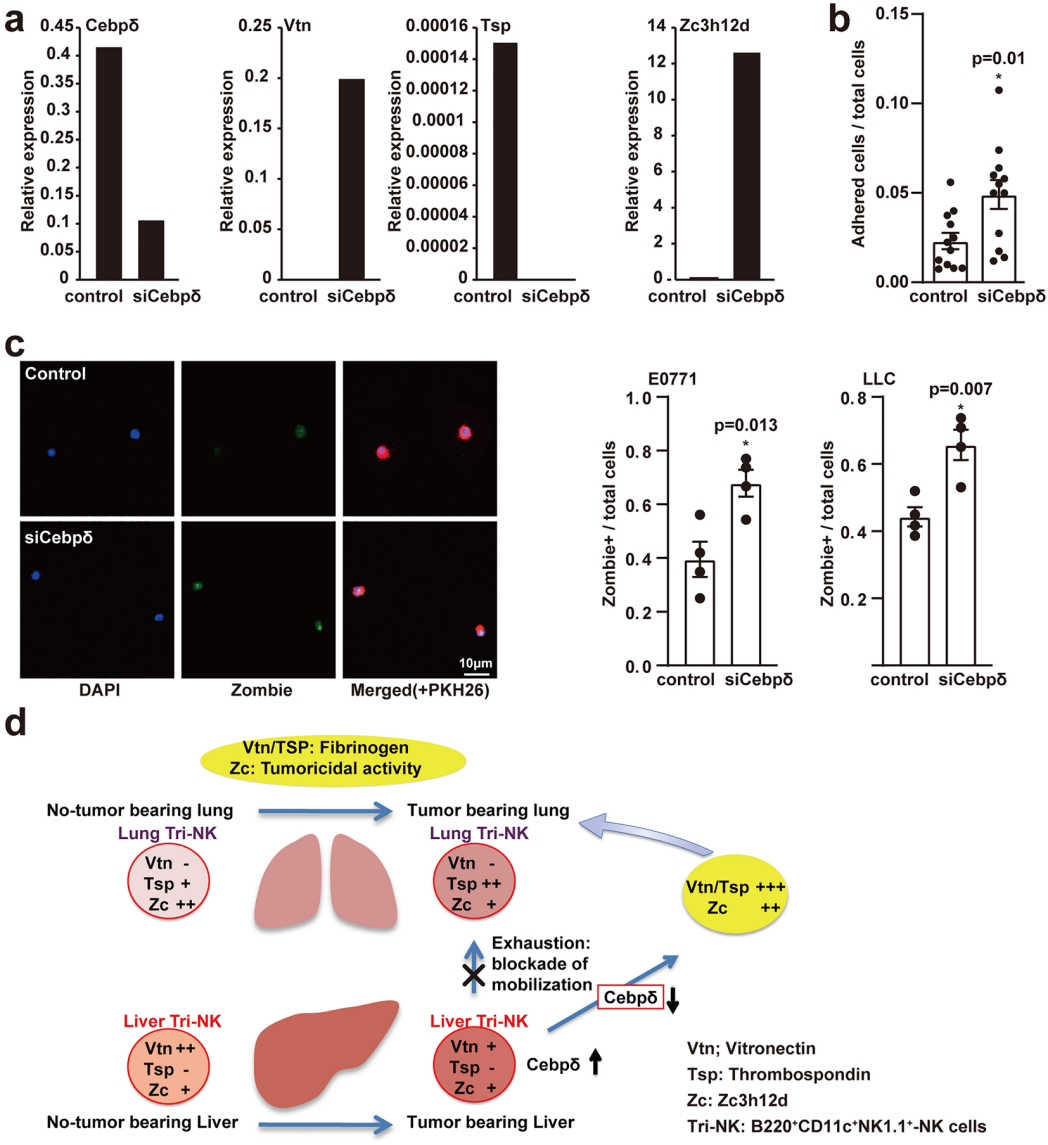
Restoration of exhaustion of Hepato-entrained NK cells by CEBPδ suppression. (**a**) mRNA levels of CEBPδ, Vtn, Tsp, and ZC3H12D in liver B220^+^CD11c^+^NK1.1^+^ NK cells derived from tumor-bearing mouse livers 48 h after siRNA administration. Repeated data in Supplementary Fig. S8. (**b**) The ratio of attached B220^+^CD11c^+^NK1.1^+^ NK cells to fibrinogen treated with control (nontarget) siRNA or CEBPδ-siRNA. (**c**) Tumoricidal assay system. Zombie green depicted representative dead tumor cells. Scale bar, 10 μm. The ratio of killed PKH-labeled tumor cells cocultured with control (nontarget) siRNA- or CEBPδ-siRNA-treated B220^+^CD11c^+^NK1.1^+^ NK cells of tumor-bearing mouse liver. N = 4. Error bars denote the mean ± SEM, and *P* values (significant) are shown in graphs (**b**, **c**). (**d**) Summary of Vtn, Tsp, and ZC3H12D expression in liver B220^+^CD11c^+^NK1.1^+^ NK cells in the pre-metastatic liver and lungs. A reduction of Vtn in the liver was seen during primary tumor progression. Intense Vtn and/or Tsp upregulation by inhibiting CEBPδ increases homing of liver B220^+^CD11c^+^NK1.1^+^ NK cells, accompanying ZC3H12D activation in killing metastatic tumor cells in the lungs.

We previously reported that a specific NK cell population, B220^+^CD11c^+^NK1.1^+^ NK cells in tumor-bearing mice, was educated in the liver and was involved in anti-metastatic function^26^. During the pre-metastatic phase, these cells perform two functions: they accumulate in the fibrinogen-rich lung area via fibrinogen-binding molecules like Vtn/Tsp and they kill circulating metastatic tumor cells that may migrate into the pre-metastatic lungs. Therefore, we focused on the liver B220^+^CD11c^+^NK1.1^+^ NK cells among CD45^+^ cells to restore anti-metastatic ability because those cells were observed the suppression of relocation from the liver to the lungs during primary tumor growth. We obtained approximately 0.3–0.5% B220^+^CD11c^+^NK1.1^+^ NK cells in tumor-bearing mouse liver CD45^+^ cells, which were then treated with control siRNA or CEBPδ-siRNA. Under the suppression of CEBPδ expression, both Vtn and ZC3H12D expression were increased in B220^+^CD11c^+^NK1.1^+^ NK cells derived from tumor-bearing mice livers 48 h after the siRNAs were administered (Fig. 5a and Supplementary Fig. S8). We then examined whether liver B220^+^CD11c^+^NK1.1^+^ NK cells from a tumor-bearing mouse could regain their binding ability to fibrinogen when the CEBPδ was knocked down. The number of CEBPδ-siRNA-treated cells but not control siRNA-treated cells increased in fibrinogen-coated plates, indicating that fibrinogen binding proteins, Vtn, and/or Tsp, were reintroduced (Fig. 5b). As previously stated, anti-metastatic NK cells should ideally be capable of killing metastatic tumor cells before infiltrating secondary lung tissues. Finally, we performed a tumoricidal assay using siRNA-treated B220^+^CD11c^+^NK1.1^+^ NK cells. Fluorescent-labeled tumor cells were cocultured with control siRNA- or CEBPδ-siRNA-treated B220^+^CD11c^+^NK1.1^+^ NK cells to calculate the number of dead tumor cells. LLC and E0771 cells were both more aggressively attacked by CEBPδ-siRNA-treated B220^+^CD11c^+^NK1.1^+^ NK cells with potent tumoricidal activity (Fig. 5c). A summary is shown in Fig. 5d; hepato-entrained B220^+^CD11c^+^NK1.1^+^ NK cells express Vtn and ZC3H12D, and a primary tumor induces a CEBPδ-dependent decrease in both molecules in those liver cells. Suppression of CEBPδ restores Vtn and ZC3H12D expression while increasing Tsp. Consequently, they may contribute to fibrinogen-rich pre-metastatic soil formation and the killing of circulating metastatic cells in the lungs.

## Discussion

A report revealed that distinct tissue environments regulate gene expression in macrophages by activating a common enhancer, PU.1, in collaboration with secondary transcription factors^27^. This outcome indicates that the tissue environment affects resident macrophages in normal tissues. NK cells are also regulated by the tissue environment. This study showed one example that NK cells changed their character depending on the environment. Isolated NK cells exhibited high levels of Vtn or Tsp expression; they show Tsp but not Vtn when they were cultured with lung tissue reproducing the lung environment, and they show Vtn but not Tsp when they were with liver tissue. Vtn and Tsp revealed tissue-specific expression patterns so it is thought that tissue-specific molecular signals emitted from the parent tissue functioned as the environmental factors. CEBPδ is a transcription factor belonging to the CCAAT/enhancer binding protein. CEBPδ is a versatile modulator of gene transcription; its physiological functions vary with cell type and cellular context, but the mechanism of environment-specific regulation is unclear^23,28^ CEBPδ also reduced tumor incidence but promoted tumor metastasis in a mouse model^23,29^. In this study, we discovered that CEBPδ is a critical transcription factor that regulates liver-specific/related molecules such as Vtn, Factor X, and albumin in the pre-metastatic situation. We also demonstrated that CEBPδ worked with a liver-specific transcription factor, Hnf1α, in the regulation of Tsp expression in the lung environment. Bioinformatical analysis of tissue-specific gene expression mechanisms unveiled that tissue specificity is primarily generated by the tissue-specific regulatory network paths but not just transcription factor expression^30^. Thus, although CEBPδ is expressed with low tissue specificity, not an NK cell-specific transcription factor, it is highly like those signaling pathways where the CEBPδ-Hnf1α axis is involved are unique to the anti-tumor NK cells. Because NK cells are very sensitive to environmental factors and these factors differ during the primary tumor progression, it is not surprising that gene expressions controlled by the CEBPδ-Hnf1α axis changed in a primary tumor size-dependent manner. Since following the increase of CEBPδ expression in the late pre-metastatic phase, the Vtn expression level of the anti-tumor NK cell was decreased, it should be the first method to knockdown CEBPδ to regain the Vtn expression. It is expected that Vtn expressed on the surface of the NK cell functions as a ligand to the fibrinogen deposition at the pre-metastatic site. The CEBPδ knockdown elevated Vtn and ZC3H12D expression and relocation of liver CD45^+^ cells in the lungs. Thus, this study showed that CEBPδ is a major regulator in the liver CD45^+^ cells to suppress their anti-metastatic abilities.

NK cell therapy has been considered a hopeful immunotherapy since the success of chimeric antigen receptor (CAR) T cell therapy. It is advantageous over CAR-T cell therapy because NK cells determine their target in an MHC-unrestricted manner and do not have a risk of graft-versus-host disease even in the cases of allotransplantation. However, due to the nature of NK cell, which is activated, or inactivated by several signaling molecules derived from local factors, therapeutical applications of NK cell are still restricted. Because the molecular mechanisms governing the NK cell anti-tumor activities are not completely understood, many investigations are still needed. Generally, immune cells equip inactivation pathways to make the cells dysfunction state. It is commonly believed that these pathways have protective roles. To avoid host tissues where an inflammatory state is lasting for a long term, an excessive immune reaction may be avoided by impairing activated immune cells. It has been reported that NK cell impairment was acquired by exhausted effector functions and upregulation of inhibitory receptors^31,32^. Also, it has been confirmed in mouse model research and cancer patients that secretion of cytokines such as IFN-γ from impaired NK cells was contained in the tumor microenvironment^33–35^. Recently, we found that the B220^+^CD11c^+^NK1.1^+^ NK cells efficiently have anti-metastatic functions evoked by extracellular mRNA through ZC3H12D signaling. Since the population of the B220^+^CD11c^+^NK1.1^+^ NK cells is very low, autografting of these cells is hard. Given the amount of B220^+^CD11c^+^NK1.1^+^ NK cells acquired from the host, it needs a long period though it is possible to increase the B220^+^CD11c^+^NK1.1^+^ NK cells in a culture dish. Furthermore, the human counterpart of the B220^+^CD11c^+^NK1.1^+^ NK cells is not fully determined. If the cells were determined or anti-metastatic NK cells were modified, it would be applicable for adoptive immunotherapy. Furthermore, knocking down CEBPδ may help to maintain the anti-metastatic ability because it will prevent tumor-associated exhaustion in NK cells.

## Methods

### Reagents

For histological analysis, the following primary antibodies (Abs) were employed: anti-vitronectin Abs (ab45139, Abcam, Cambridge, MA, and MAB38751, R&D Systems, Inc., Minneapolis, MN), anti-thrombospondin 1 Abs (Ab-11, Thermo Fisher Bio-scientific, Hudson, NH, and ab1823, Abcam), anti-CD45 Abs (ab10558, Abcam, and M0701, DAKO, Carpinteria, CA, USA). Additionally, for B220^+^CD11c^+^NK1.1^+^ NK cell sorting, the following antibodies were used; PE/Cy7 anti-mouse B220 (103222, BioLegends), PE anti-mouse CD11c (117308, BioLegends), APC anti-mouse NK1.1 (108710, BioLegends), and those isotype control antibodies, PE/Cy7 Rat IgG2a, κ (400521, BioLegends), PE American Hamster IgG (12-4888-81, Thermo Fisher), APC Mouse IgG2a κ (550882, BD Biosciences).

### Animals

C57BL6 mice were bought from Clea Japan (Tokyo, Japan) or SLC (Shizuoka, Japan). We conducted in vivo and in vitro experiments using C57BL6 mice. These mice were kept in the Animal facilities of Shinshu University and Tokyo Women’s Medical University under specific pathogen-free conditions. All experiments were conducted under institutional guidelines (No. 021029-1: Shinshu University) and (No. 13-80, 14-80, 15-126, AE16-107, and AE17-59-3-B: Tokyo Women’s Medical University), which were authorized by the Animal Experimentation Committees of Shinshu University and Tokyo Women’s Medical University, respectively. The institutional guidelines comply with the ARRIVE guidelines.

### Tumor cell lines and tumor-conditioned medium (TCM)

Dr. Sirotnak (Memorial Sloan–Kettering Cancer Center, New York, NY) established the E0771 breast cancer cell line, and Dr. Mihich (Roswell Park Memorial Institute, Buffalo, NY) kindly provided it^36^. The C57BL/6 mouse-syngeneic LLC cell line and E0771 cells were maintained in Dulbecco’s modified Eagle’s medium (DMEM) supplemented with 10% fetal bovine serum (FBS), 1% penicillin–streptomycin mixed solution (Nacalai Tesqu, Kyoto, Japan). To obtain TCM, we incubated the cells overnight in a serum-free medium.

### Experimental metastatic model

The experiments were conducted as the methods described in our prior studies^5,16,17^. The period when the distant primary tumor grew was deemed the pre-metastatic phase. The metastatic phase was defined as the period after the intravenous (i.v.) injection of tumor cells into tumor-bearing mice. Synergistic tumor grafts were generated through subcutaneous (s.c.) or mammary fat pad (m.f.p.) implantation of 5 × 10^6^ tumor cells into 8 to 10-week-old mice. We injected tumor cells into mice with growing primary tumors of the same size (size-matched). For the tumor cell homing assays, 1–2 × 10^4^ fluorescent dye (PKH26, Sigma-Aldrich, St. Louis, MO, USA)-labeled metastatic cells were i.v. infused into the mice. At 24–48 h after tumor cell infusion, the lungs were perfused with phosphate-buffered saline (PBS) under physiological pressure to remove circulating tumor cells and then excised. Four to five randomly selected lung tissue fragments (3 mm in diameter) were chosen, and three 10 μm sections per fragment were evaluated using a confocal microscope (SP8 Leica, LSM-710, Carl Zeiss MicroImaging GmbH, Germany) or a fluorescence microscope (BZ-9000, Keyence, Osaka, Japan). The labeled tumor cell counts were normalized to the total tissue surface area. Age- and sex-matched mice were used for the experiments.

### Isolation of CD45^+^ cells and B220^+^CD11c^+^NK1.1^+^ NK cells

The experiments were performed as the methods described in our prior studies^16,17^. To obtain tissue containing CD45^+^ cells, we digested mouse livers, and lungs with 0.5 mg/mL of collagenase, 1 mg/mL of dispase, and DNase at 37 °C for 45 min. The cell suspensions were incubated with mouse CD45-Microbeads (MACS, Miltenyi Biotec, Auburn, CA, USA), and the captured cells were used for in vitro culture, siRNA application, or in vivo injection.

B220^+^CD11c^+^NK1.1^+^ NK cell isolation from the tumor-bearing liver was conducted as follows. First, eight-week-old mice were subcutaneously injected in the back with 20 μL of LLC cells (5 × 10^4^/μL) for tumor-bearing. Then, the mice were sacrificed when a tumor with a diameter of 9 mm grew, and the liver was cut out. The liver was treated with collagenase, dispase, and DNase as above. After pipetting and filtration with a cell strainer, the cell suspension had the removal of significant liver cells with HISTOPAQUE1083 (Thermo Fisher Bio-scientific, Hudson, NH, USA). The resulting cells containing immune cells were then electroporated with Nucleofector Technology, Mouse T cell Nucleofector™ Kit, program No. X-001 (Amaxa, Lonza, Basel, Switzerland), and siRNAs (nontarget or CEBPδ) (Dharmacon, GE Healthcare, Lafayette, CO, USA). The pulsed cells were then labeled with APC-anti-NK1.1 antibody, PE-anti-CD11c antibody, PE/Cy7-anti-B220 antibody, and isotype control antibodies of those and subjected to FACS with Aria III (BD Biosciences, San Jose, CA, USA) to isolate B220^+^CD11c^+^NK1.1^+^ NK cells. The sorted cells were cultured at 37 °C and 5% CO_2_ with a growth medium comprising HPLM (Thermo Fisher Bio-scientific, Hudson, NH, USA), 1% FBS, 100U/mL recombinant human interleukin 2, and 50 μg/mL gentamicin.

### CD45^+^ cell culture system using organ tissues

The experiments were conducted as the systems described in our prior study^16^. In the organ culture experiments, 2 mm^2^ tissue specimens were cultured in DMEM without or with FCS (1%) in an upper well culture insert (400 nm pores). Additionally, CD45^+^ cells isolated from organs were cultured in the lower wells and activated by organ tissues in the upper wells for 24–48 h.

### Gene knockdown by siRNA

For the gene silencing experiments, cells were transfected with siRNA for Foxa1, Foxa2, Foxa3, Hnf1a, Hnf4a, CEBPa, CEBPd, GATA6, NF-kappaB (Rela), Sp1, Ap2a, or nontargeting siRNA using Nucleofector Technology (Amaxa, Lonza, Basel, Switzerland) or the Accell siRNA transfection method (Dharmacon, GE Healthcare, Lafayette, CO, USA). The siRNA sequences are demonstrated in the Supplementary Methods.

### Immunohistochemistry and cell counts

The experiments were conducted as the methods described in our prior studies^5,16,17^. To immunostain the frozen sections, we used anti-Vtn, and anti-Tsp. For negative control staining, we used isotype-matched IgG. The immunostained cell area values are indicated as the number of pixels normalized to the DAPI signals. Labeled tumor cells were found by confocal microscopy or fluorescence microscopy. The total surface area normalizes the number of tumor cells.

### Microarray analysis

Microarray screening of photoconverted KikGR cells from the liver and lung tissue using a *GeneChip Mouse Genome 430 2.0 Array* (*Affymetrix*) was previously described^16^.

Microarray data are deposited in NCBI; Supplementary Table S1—GSE76235.

### Quantitative PCR

Total RNA isolation, complementary DNA synthesis, quantitative PCR with TaqMan Fast Advanced Master Mix (Applied Biosystems, Foster City, CA, USA) and a PCR detector StepOnePlus (Applied Biosystems), and results assessment were conducted as the procedures described in previous our studies^5,16,17^. In this study, gene expression of Vtn, Tsp, Foxa1, Foxa2, Foxa3, Hnf1α, Hnf4α, CEBPα, CEBPδ, Gata6, NFκB (Rela), Sp1, Ap2a, and Zc3h12d were analyzed. The primer information is described in the Supplementary Methods.

### Attachment assay to fibrinogen

The B220^+^CD11c^+^NK1.1^+^ NK cells were resuspended in the growth medium described above at 20–25 cells/μL. The 20 μL of suspension was then placed on the fibrinogen-coated cell culture dish and made an approximately 3-mm-diameter dome-like culture condition. After incubation for 90 min at 37 °C and 5% CO_2_, the cells are fixed with 4% paraformaldehyde (Nacalai Tesque, Kyoto, Japan) at RT and washed with PBS. The resulting adhered cells were then counted. Six drops were evaluated for 1 B220^+^CD11c^+^NK1.1^+^ NK cell isolation, and 12 drops from two isolations were examined. The fibrinogen-coating was conducted as follows. First, fibrinogen was diluted to 100 ng/mL with PBS and plated on culture dishes. Then, after incubation at 4 °C O/N, the dishes were washed with PBS.

### Tumoricidal assay

E0771 cells or LLC cells were plated on 24 well plates and at 50% confluent, nontarget- or CEBPδ-siRNA-treated B220^+^CD11c^+^NK1.1^+^ NK cells (post pulse 24 h) were included. After 24 h of cultivation with a growth medium for B220^+^CD11c^+^NK1.1^+^ NK cells, the cells were fixed with 4% paraformaldehyde and washed with PBS. The adhered cells were stained with Zombie Green™ Fixable Viability Kit (BioLegend) and DAPI at RT in the darkness. After washing with PBS, resulting Zombie-fluorescent positive cells were counted. For each well, 20–40 DAPI-positive cells were evaluated, and the values were defined as Zombie-positive cells/total cells. Two wells of E0771 or LLC cells added with each RNAi-governed B220^+^CD11c^+^NK1.1^+^ NK cell were assessed for one isolation of the B220^+^CD11c^+^NK1.1^+^ NK cells. Totally four wells were examined with two isolations of the B220^+^CD11c^+^NK1.1^+^ NK cells.

### Statistical analysis

Data are indicated as mean ± SEM. Student’s *t*-test and analysis of variance were used for comparison between two samples and among more than three samples, respectively. *P* values of < 0.05 were considered statistically significant; * are placed beside actual *P* values in graphs. All plot data were assessed with GraphPad Prism ver. 8.

## Supplementary Information

Supplementary Information 1.

Supplementary Information 2.

## Data Availability

Microarray data were deposited in the NCBI Gene Expression Omnibus as GSE76235 (Supplementary Table S1). The raw data used in this study are available from the corresponding author upon request.
