# Supplementary material for: Suppression of CEBPδ recovers exhaustion in anti-metastatic immune cells
Source: Sci Rep. 2023 Mar 8;13:3903. doi: 10.1038/s41598-023-30476-4 (PMC9995318; doi:10.1038/s41598-023-30476-4)
Supplement: Supplementary file 1 — Supplementary Information 1. [file 41598_2023_30476_MOESM1_ESM.pdf]

Suppression of CEBPδ recovers exhaustion in anti-metastatic immune cells

Chenxue Yin, Masayoshi Kato, Takeshi Tomita, Yibing Han, Sachie Hiratsuka

Gene symbol	Lung	Liver	Fold change
Cebpd	110	5	3.38
Gata6	86	19	1.23
Foxa2	18	4.72	1
Sp1	3269	2447	-0.4
Fos	5999	6119	-0.95
Cebpa	557	608	-1.03
Ap2a1	106	130	-1.19
Hnf1a	69	89	-1.2
Foxa1	1	1.75	-1.72
Jun	3392	6040	-1.73
Ap2a2	67	857	-4.5
Rela	28	371	-4.6
Foxa3	5.2	162	-5.8
Hnf4a	1	126	-7.8
Vtn	8.8	3053	-9.3
Thbs1(Tsp1)	696	12	4.89
Zc3h12d	1373	10	6.19

The list of putative transcription factors related to Vtn and Tsp1
Liver $CD45^{+}$ cells relocated in the lungs and stayed in the livers obtained from tumor-conditioned media-stimulated mice were compared. The fold changes of mRNA levels in the lung versus the liver are revealed. The data are deposited in GSE 76235.

Table S1

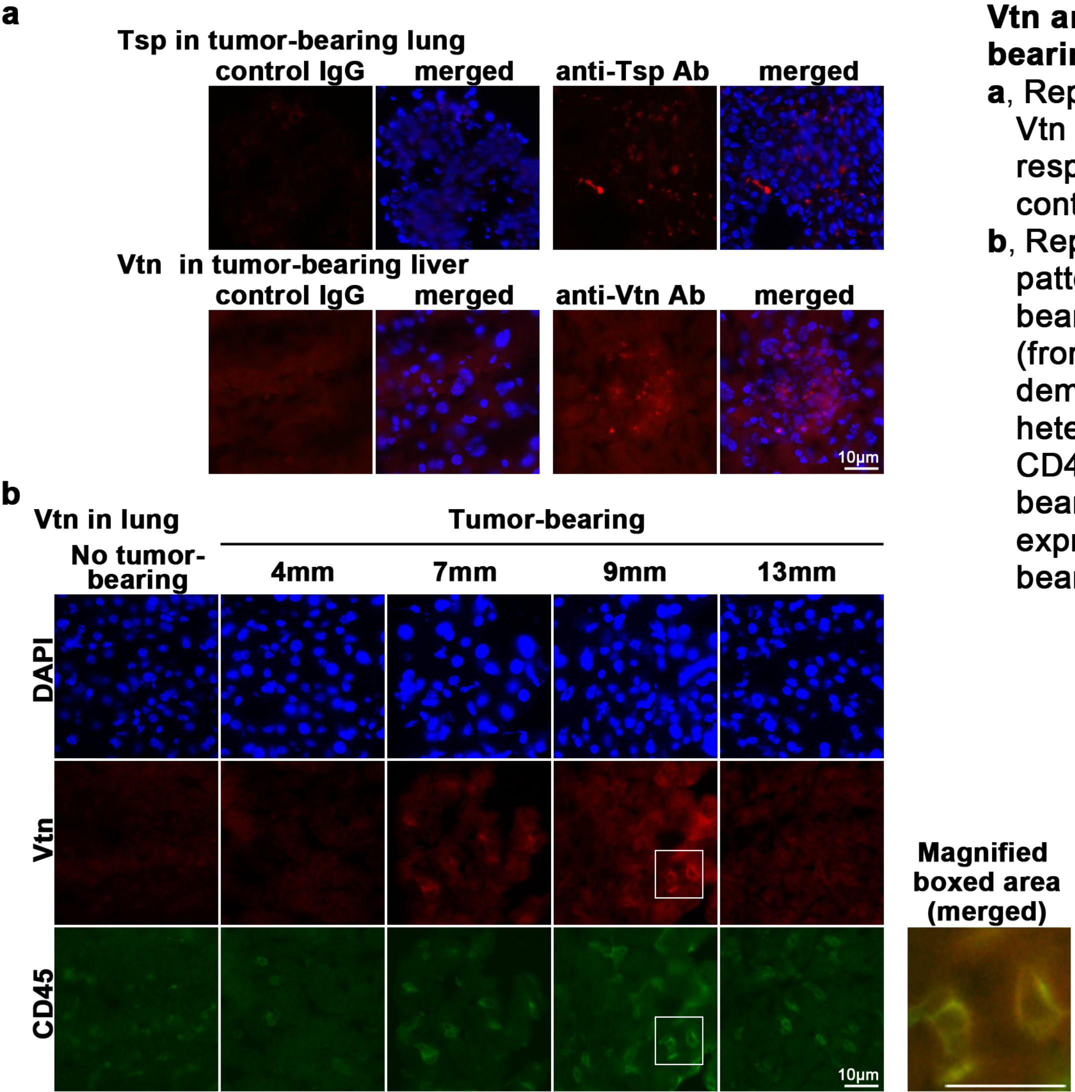

Vtn and Tsp expressions in tumor-bearing lung and livers

a, Representative staining of Tsp, and Vtn in tumor-bearing lung and livers respectively, when compared with control IgG.

b, Representative Vtn expression pattern in $CD45^+$ cells in tumor-bearing lungs. Primary tumor size (from no tumor to 13 mm) was demonstrated. An increase in heterogeneous expression of Vtn in $CD45^+$ cells was revealed in tumor-bearing (9 mm) lungs. However, that expression was decreased in tumor-bearing (13 mm) lungs. Bars: 10 μm

Figure S1

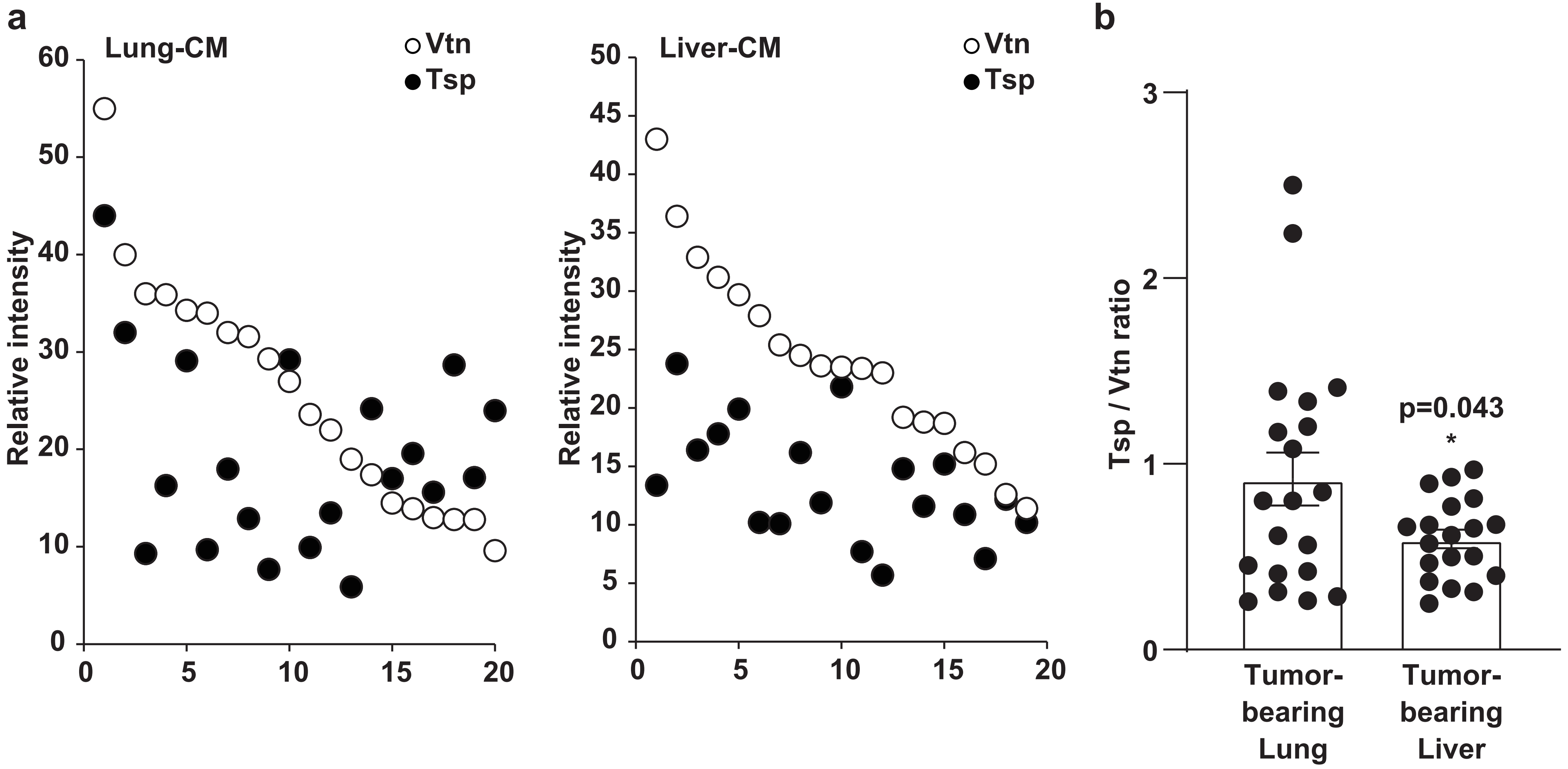

The Increase of Tsp expression in liver Vtn+CD45+ cells cultured with tumor-bearing lung tissues

a. Vtn and Tsp relative expression levels in liver Vtn+CD45+ cells defined by immunostaining. The perpendicular axis indicates signal intensities of Vtn (open circle) and Tsp (filled circle) in liver Vtn+CD45+ cells cultured with conditioned medium (CM) (24 h) derived from the lung (left panel) or the liver (right panel) of tumor-bearing mice. The horizontal axis indicates cell number. Each value was normalized by DAPI immunostaining.

b. Comparison of Tsp/Vtn ratios. The graph shows averages of the Tsp/Vtn ratios, displayed in a, of lung-CM or liver-CM-treated cells.

Figure S2

Figure S3

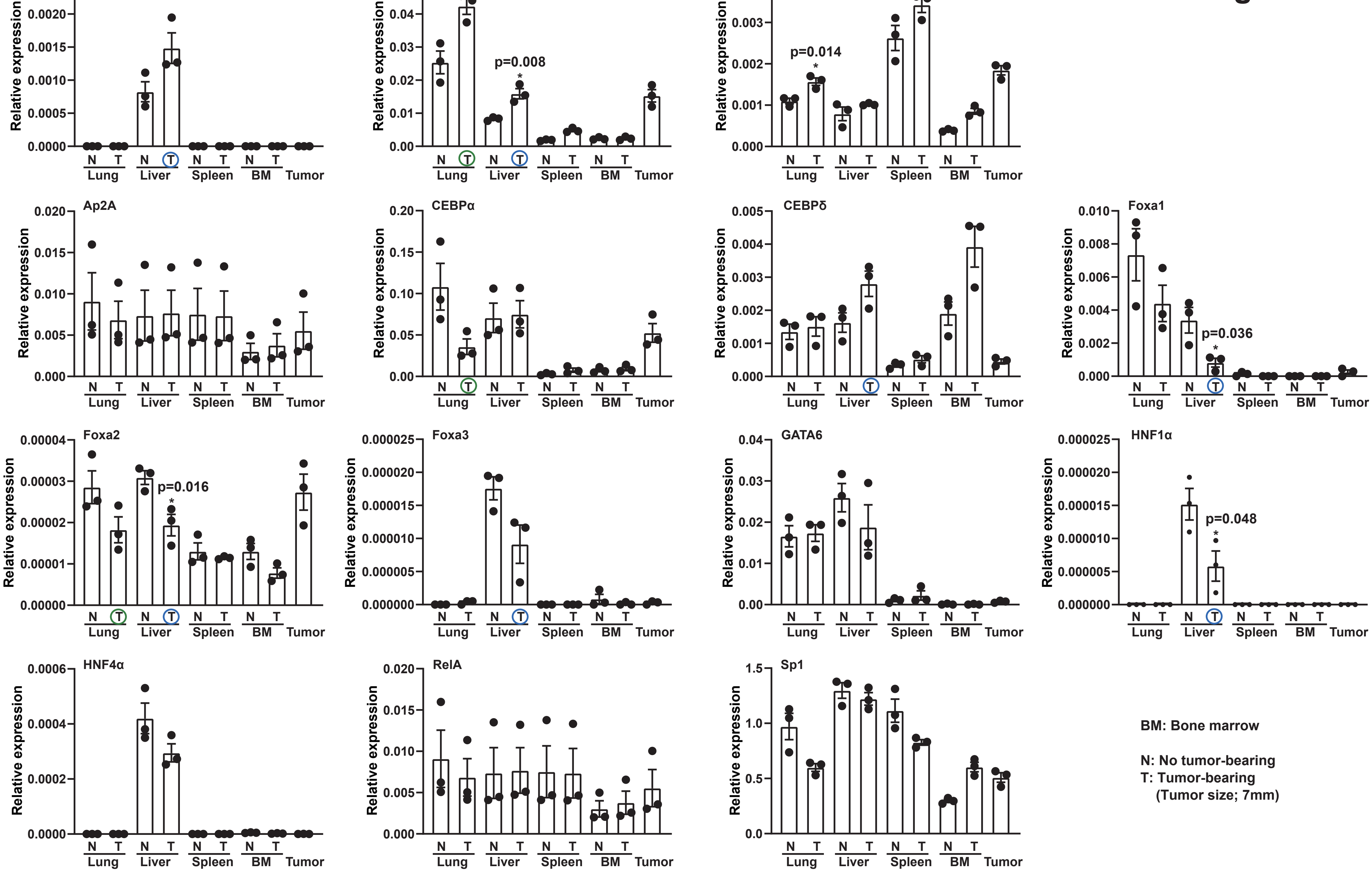

Data related to heatmaps in Fig.2b mRNA levels of Vtn, Tsp, Zc3h12d, and different liver-related transcription factors in primary tumor tissue and various organs including lung, liver, spleen, and bone marrow derived from tumor-free (N) or E0771-bearing (7mm, T) mice. Total RNAs were purified from indicated tissues and cDNAs were prepared. The cDNAs were then assessed by quantitative PCR. Blue and green circles are shown 1.5 fold change between (N) vs (T). Error bars represent the mean ± SEM, and P-values (significant) are shown in graphs. The relative mRNA levels were normalized by β-actin. N = 3

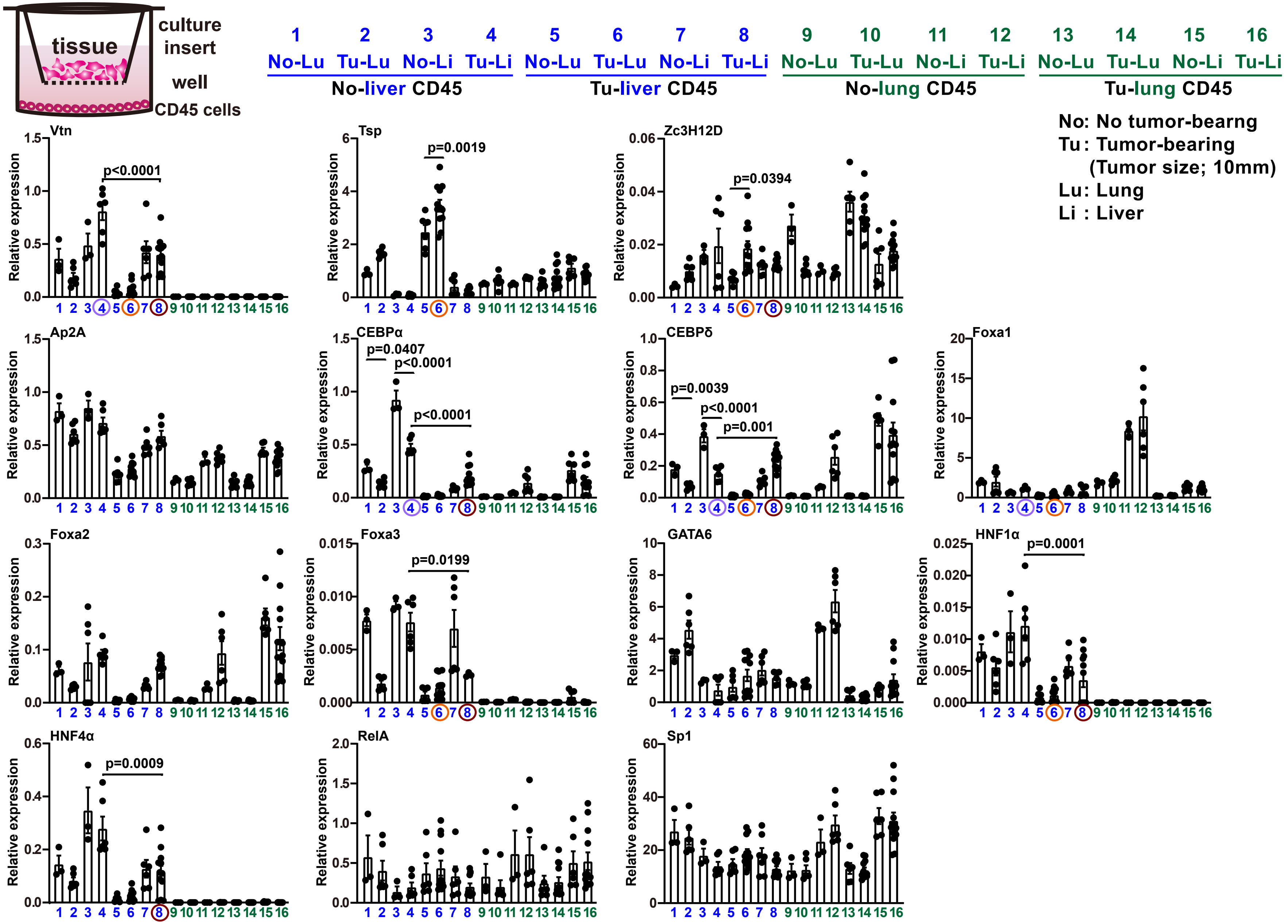

Data related to heatmaps in Fig.2b mRNA levels in CD45+ cells in lower wells cultured with organ tissues in upper well. Numbers show multiple combinations of CD45+ cells and organ tissues obtained from tumor-free or E0771-bearing mice (tumor size: 10mm). Total RNAs were purified from indicated conditioned CD45+ cells and cDNAs were prepared. The cDNAs then were assessed by quantitative PCR. Error bars represent the mean ± SEM, and P-values (significant) are shown in graphs. The relative mRNA levels were normalized by β-actin. N = 3-12

Figure S4

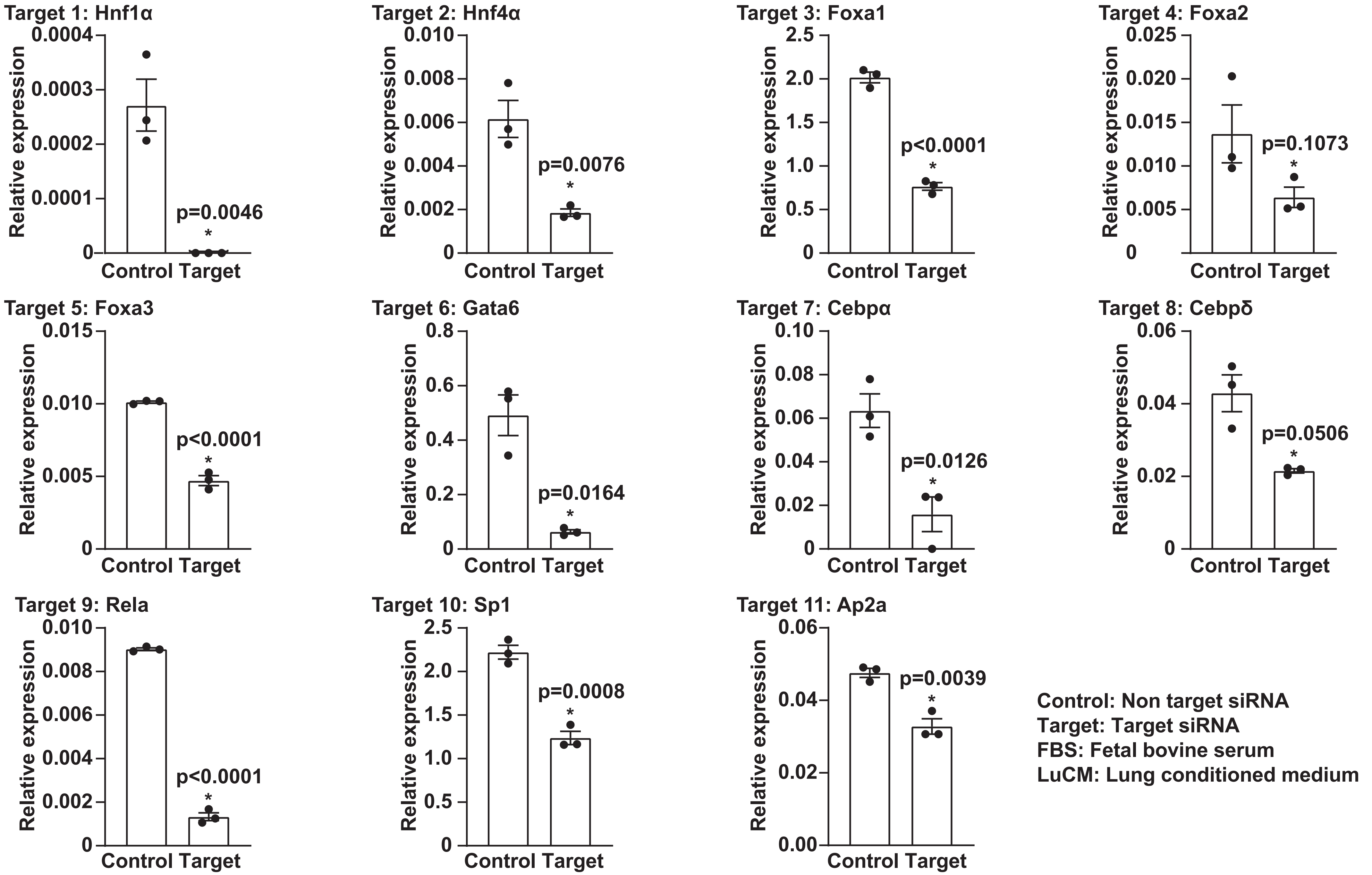

Validation of siRNAs

Target siRNAs were transfected by either Accell or Nucleofector method into liver $CD45^+$ cells. Reductions of target mRNA expression were confirmed by qPCR. Liver $CD45^+$ cells were cultured either in the presence or absence of lung conditioned medium. Total RNAs were extracted from the cells 24-48 h after the transfection, and cDNAs were prepared. The cDNAs then were assessed by quantitative PCR. Error bars represent the mean ± SEM, and P-values (significant) are shown in graphs. The relative mRNA levels were normalized by β-actin. N = 3

Figure S5

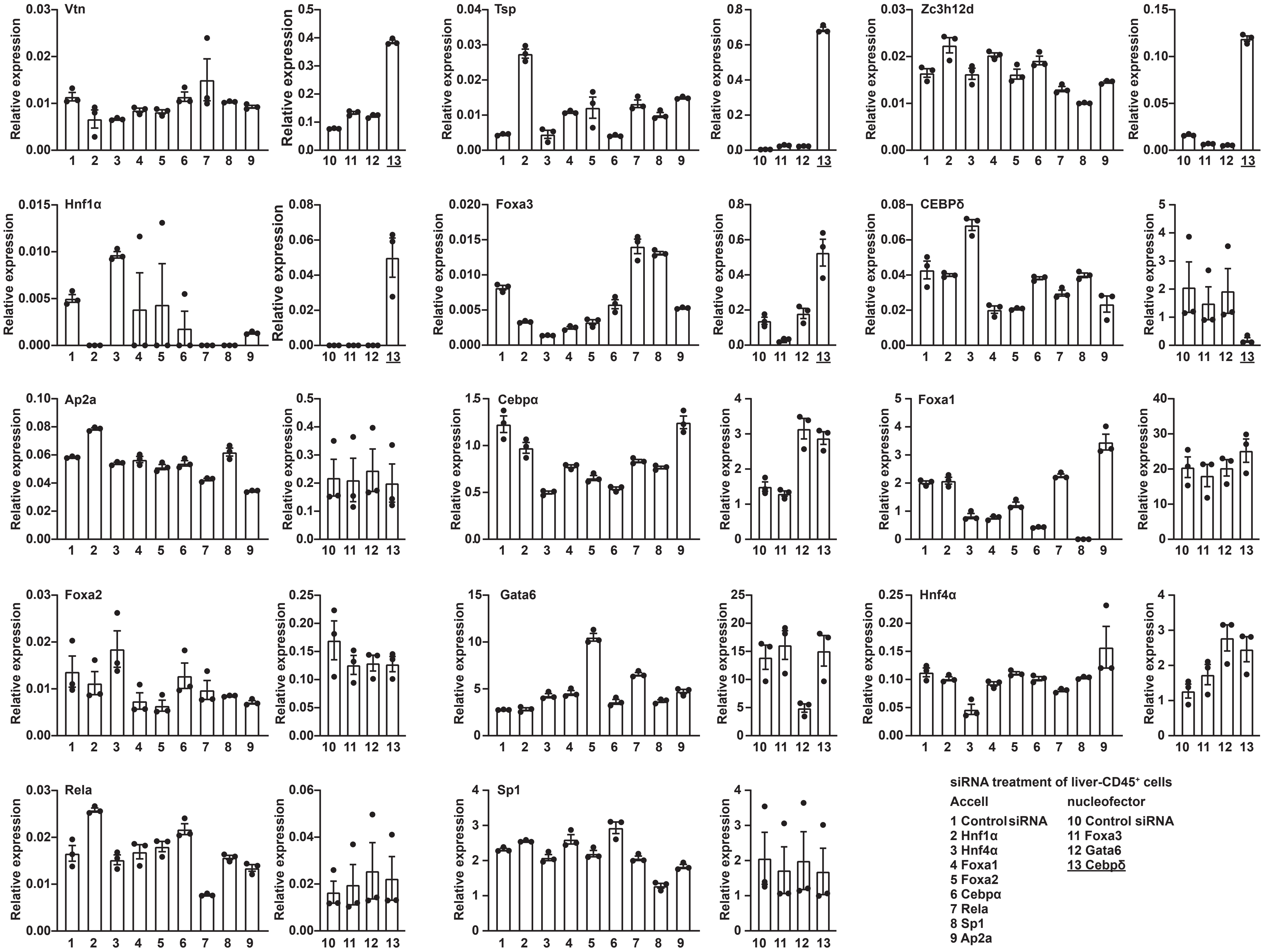

Several gene expressions in liver CD45$^+$ cells treated with siRNAs. Data are related to Fig.3a.
The siRNA delivery systems were used for each siRNA (transfection with “Accell” and electroporation with “nucleofector”) and evaluated with nontarget siRNA. Numbers show several indicated siRNAs. Total RNAs were purified from indicated siRNA-introduced CD45$^+$ cells and cDNAs were prepared. The cDNAs were then evaluated by quantitative PCR. Error bars represent the mean ± SEM, and P-values (significant) are shown in graphs. The relative mRNA levels were normalized by β-actin. N = 3

Figure S6

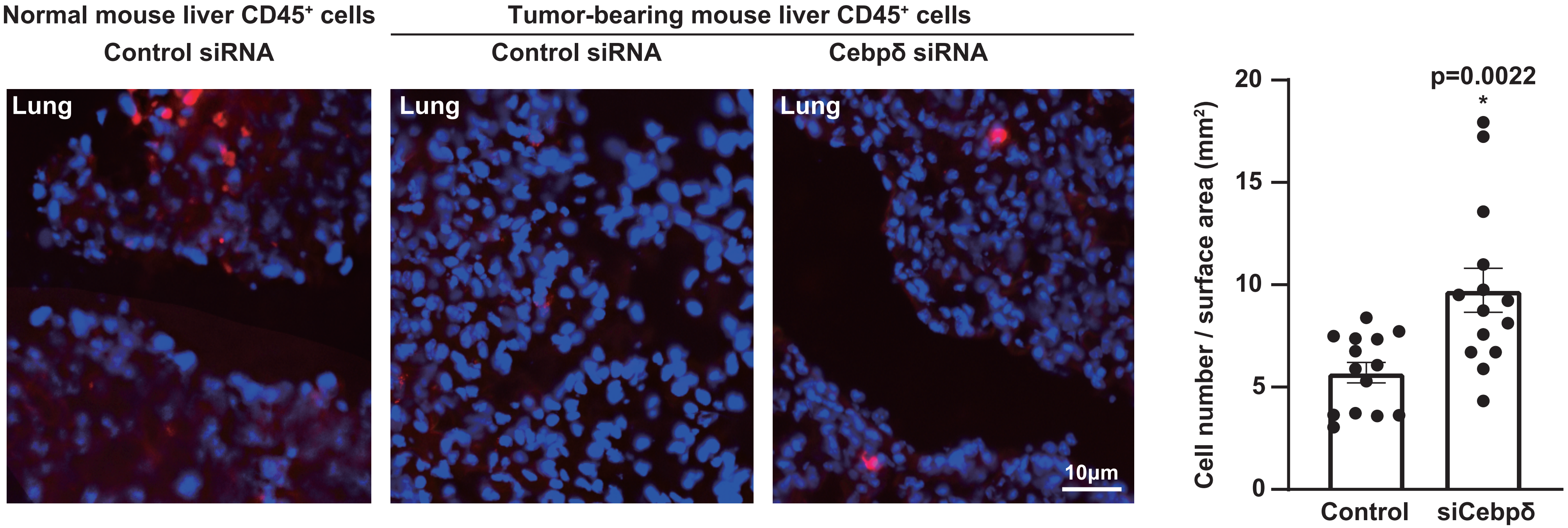

Number of accumulated control (nontarget) siRNA- or Cebpδ siRNA-treated CD45⁺ liver cells in tumor-bearing mouse lungs
Liver CD45⁺ cells derived from E0771-bearing mice were used with control siRNA- or Cebpδ siRNA. Fluorescent-labeled those cells were injected into E0771-bearing mice and counted 24 h after injection. N=6 (2 lobes in one mouse) Scale bar: 10 μm

Figure S7

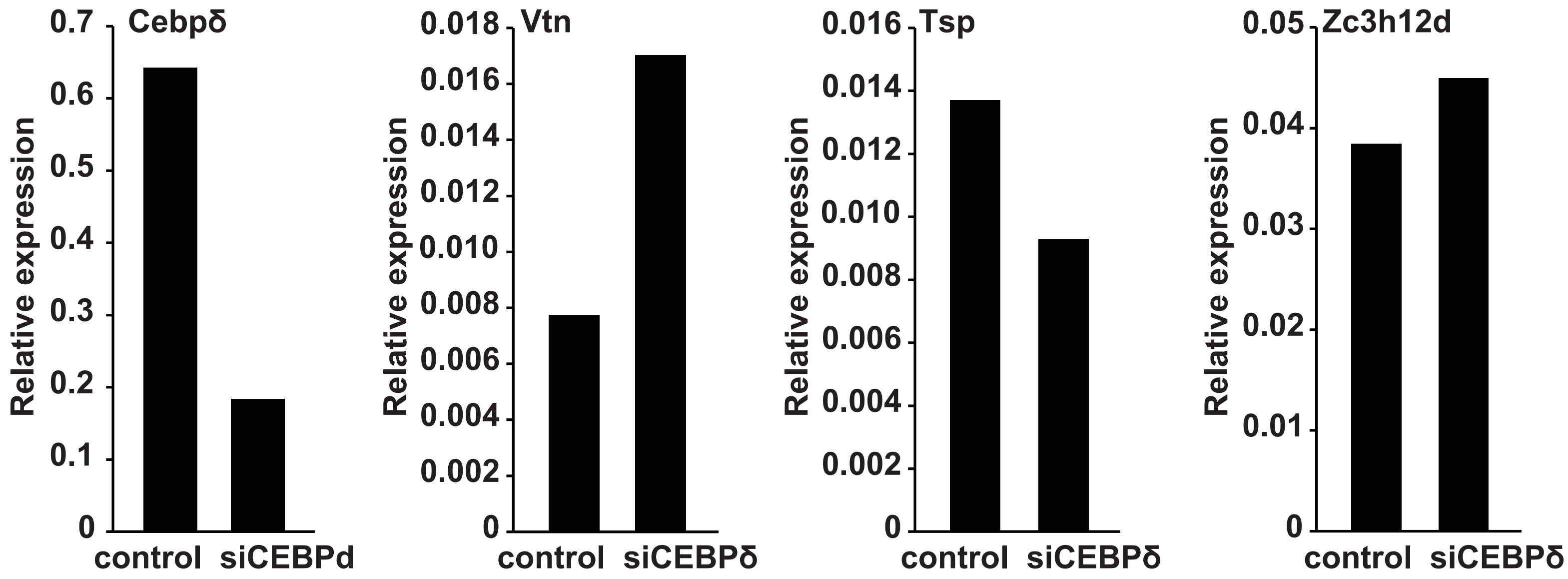

mRNA levels of Cebpδ, Vtn, Tsp, and Zc3h12d in $B220^{+}CD11c^{+}NK1.1^{+}$NK cells from tumor-bearing mouse liver 48 h after siRNA administration, the mRNA level of the cells were examined by quantitative PCR.

Figure S8
