# Supplementary material for: Suppression of CEBPδ recovers exhaustion in anti-metastatic immune cells
Source: Sci Rep. 2023 Mar 8;13:3903. doi: 10.1038/s41598-023-30476-4 (PMC9995318; doi:10.1038/s41598-023-30476-4)
Supplement: Supplementary file 2 — Supplementary Information 2. [file 41598_2023_30476_MOESM2_ESM.docx]

**Supplementary methods**

**siRNA sequences**

Hnf3a, NM_008259

siRNA sense: 5′- CGGGUUUCAUUAUUAUUCC-3′

siRNA antisense: 5′-GGAAUAAUAAUGAAACCCG-3′

Hnf3b, NM_010446

siRNA sense: 5′-CCUCCCUACUCGUACAUCU-3′

siRNA antisense: 5′-AGAUGUACGAGUAGGGAGG-3′

Gata6, NM_010258

siGENOME Set of 4 MQ-065585-01-0002

Hnf1a, NM_009327

siRNA sense: 5′-CACAAGUUGGCCAUGGACACCUAUA-3′

siRNA antisense: 5′-UAUAGGUGUCCAUGGCCAACUUGUG-3′

Hnf4a, NM_008261

siRNA sense: 5′-AGAGGUCCAUGGUGUUUAA-3′

siRNA antisense: 5′-UUAAACACCAUGGACCUCU-3′

Cebpa, NM_007678

siRNA sense: 5′-GCAAAAAUGUGCCUUGAUAUU-3′

siRNA antisense: 5′-AAUAUCAAGGCACAUUUUUGC-3′

Sp1, NM_013672

siRNA sense: 5′-GGAACAGAGUGGCAACAGU-3′

siRNA antisense: 5′-ACUGUUGCCACUCUGUUCC-3′

Ap-2a, NM_007459

siRNA sense: 5′-GAGCAUGUGCACGCUGGCCA-3′

siRNA antisense: 5′-UGGCCAGCGUGCACAUGCUC-3′

Cebpd, NM_007679

siGENOME Set of 4 MQ-060294-01-0002

NF-kappaB, NM_009045

siRNA sense: 5′-CTCAAGATCTGCCGAGTAA-3′

siRNA antisense: 5′-UUCUCGGCGUCUUGG-3′

Foxa3, NM_008260

siGENOME Set of 4 MQ-046244-01-0002

**TaqMan assay primer and probe sets**

HNF3a, NM_008259; Mm00484713_m1

HNF3b, NM_010446; Mm01976556_s1

GATA6, NM_010258; Mm00802636_m1

HNF1a, NM_009327; Mm00493434_m1

Hnf4a, NM_008261; Mm01247712_m1

Cebpa, NM_007678; Mm00514283_s1

Sp1, NM_013672; Mm00489039_m1

Ap2a, NM_007459; Mm01279159_m1

Cebpd, NM_007679; Mm00786711_s1

NF-kappaB, NM_009045; Mm00501346_m1

Foxa3, NM_008260; Mm00484714_m1

Vtn, NM_011707; Mm00495976_m1

Tsp, NM_011580; Mm00449032_g1

Zc3h12d, NM_172785; Mm01191870_m1
